# Additional squamosal suture synostosis and segmented intracranial volume in patients with non-syndromic sagittal synostosis

**DOI:** 10.1007/s00381-018-04029-4

**Published:** 2019-01-07

**Authors:** Junnu Leikola, Arja Heliövaara, Mika Koivikko, Virve Koljonen

**Affiliations:** 1 Cleft Palate and Craniofacial Center, Department of Plastic Surgery, Töölö Hospital, Helsinki University Central Hospital, P.O. Box 266, FI-00029 Helsinki, Finland; 2 Helsinki Medical Imaging Center, Department of Radiology, Helsinki University Central Hospital, Helsinki, Finland; 3 Department of Plastic Surgery, University of Helsinki and Helsinki University Hospital, Helsinki, Finland

**Keywords:** Squamosal suture, Intracranial volume, Craniosynostosis, Scaphocephaly

## Abstract

**Purpose:**

To evaluate the incidence of squamosal suture synostosis (SQS) in children with non-syndromic sagittal synostosis and to evaluate whether the additional SQS affects the intracranial volume (ICV).

**Methods:**

Thirty-four consecutive patients (23 boys) who had been operated by cranial vault remodelling because of sagittal synostosis were compared retrospectively from 3D-CT imaging data sets obtained from volumetric CT. The mean age of the patients at preoperative CT imaging was 0.48 (range 0.13–1.3) years. Mann-Whitney *U* test was used in the statistical analyses.

**Results:**

Sagittal synostosis was combined with SQS in four children (11.7%) but the additional SQS did not affect the ICV. SQS was unilateral in all children, two were located on the right and two on the left side. The length of the SQS varied between 4 and 27 mm. The children with SQS had a shorter sagittal suture synostosis length ratio (length of synostosis / total sagittal suture length × 100) than those without SQS.

**Conclusions:**

The incidence of SQS in non-syndromic sagittal synostosis was 11.7% but SQS did not affect the ICV.

## Introduction

Squamosal suture is a minor lateral skull suture that separates the parietal and squamous temporal bones. In the recent literature, the squamosal suture synostosis (SQS) has gained growing research interest [1, 2, 4–6]. However, little is known about the incidence, phenotype, or management of SQS [6].

Two large retrospective single-centre reviews on the incidence of SQS in all types of craniosynostosis patients have reported incidences of 9% [2] and 20.8% [4]. SQS is more common in patients with syndromic and complex craniosynostosis [1, 2, 4], and the incidence seems to increase with increasing age [2]. When present with a single major suture fusion, SQS has been most commonly associated with non-syndromic coronal synostosis [2, 4].

The aim of this study was to evaluate the incidence of SQS in children with non-syndromic sagittal synostosis. We also evaluated whether the additional squamosal suture synostosis affects the intracranial volume.

## Patients and methods

The study protocol was approved by Helsinki University Central Hospital. Principles outlined in the Declaration of Helsinki were followed.

For this retrospective study, a cohort of 34 non-syndromic sagittal synostosis patients with operated non-syndromic isolated sagittal synostosis was used. The cohort consisted of 23 (68%) boys and 11 (32%) girls. The mean age at the preoperative imaging was 0.48 (range 0.13–1.3) years.

All cranial CTs were obtained using a 64-slice scanner (LightSpeed VCT, GE Medical Systems, Milwaukee, WI, USA) with the following parameters: Helical Full, 0.5 s rotation time, increment 39.37 mm/rotation (pitch 0.984:1), 100 kV 40 mA and 120 kV 50 mA tube current for those aged less than and over 1.5 years, respectively. Images were reconstructed 0.625-mm thick at 0.312-mm intervals.

The preoperative CT images were reviewed by an experienced radiologist (MK) focusing specifically on squamosal sutures. We also recorded the laterality of squamosal suture synostosis and the length of the synostosis. Preoperative segmented intracranial volumes were calculated [3]. Mann-Whitney *U* test was used in the statistical analyses.

## Results

**Fig. 1:**
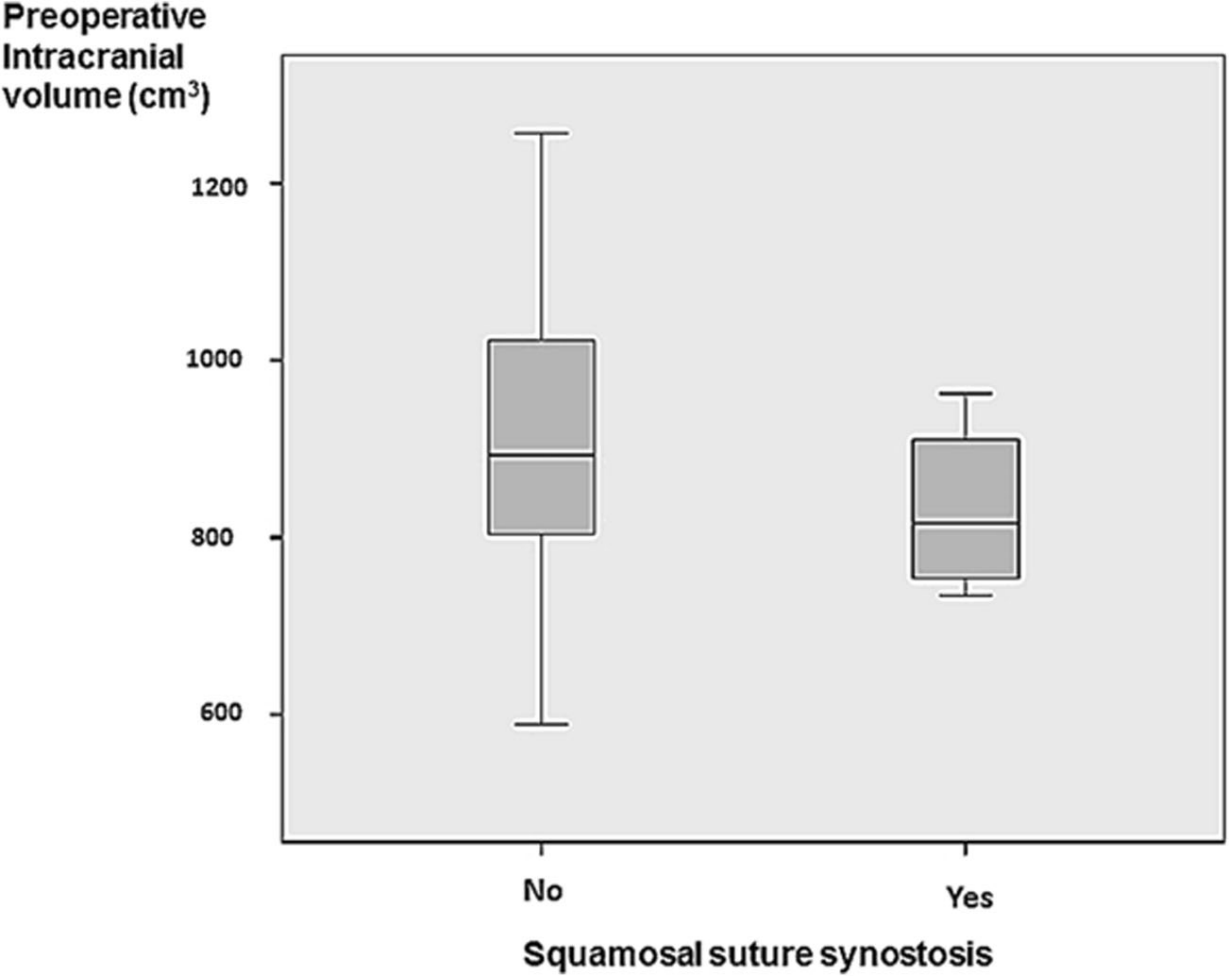
Box-plot displaying preoperative intracranial volume in children with sagittal suture synostosis with and without squamosal suture synostosis

Sagittal synostosis was combined with SQS in four children (11.7%) Table 1. The additional SQS did not affect the ICV, *p* = 0.285 (Fig. 1). The mean ICV in children without SQS was 907.80 cm^3^ (SD 28.09), and the mean of those with SQS was 832 cm^3^ (SD 50.45).

**Table 1:** Patient data of the four children with combined squamosal suture synostosis (SQS) and sagittal synostosis

Patient number	Age at imaging, months/gender	Laterality of SQS	Length of SQS (mm)	Location of SQS	Location of sagittal suture synostosis
1	1.5/male	Right	10	Middle third	Posterior
2	2.3/male	Left	4	Anterior third	Posterior
3	5.7/female	Left	12	Anterior third	Middle
4	6.9/male	Right	27	Posterior third	Posterior

**Fig. 2:**
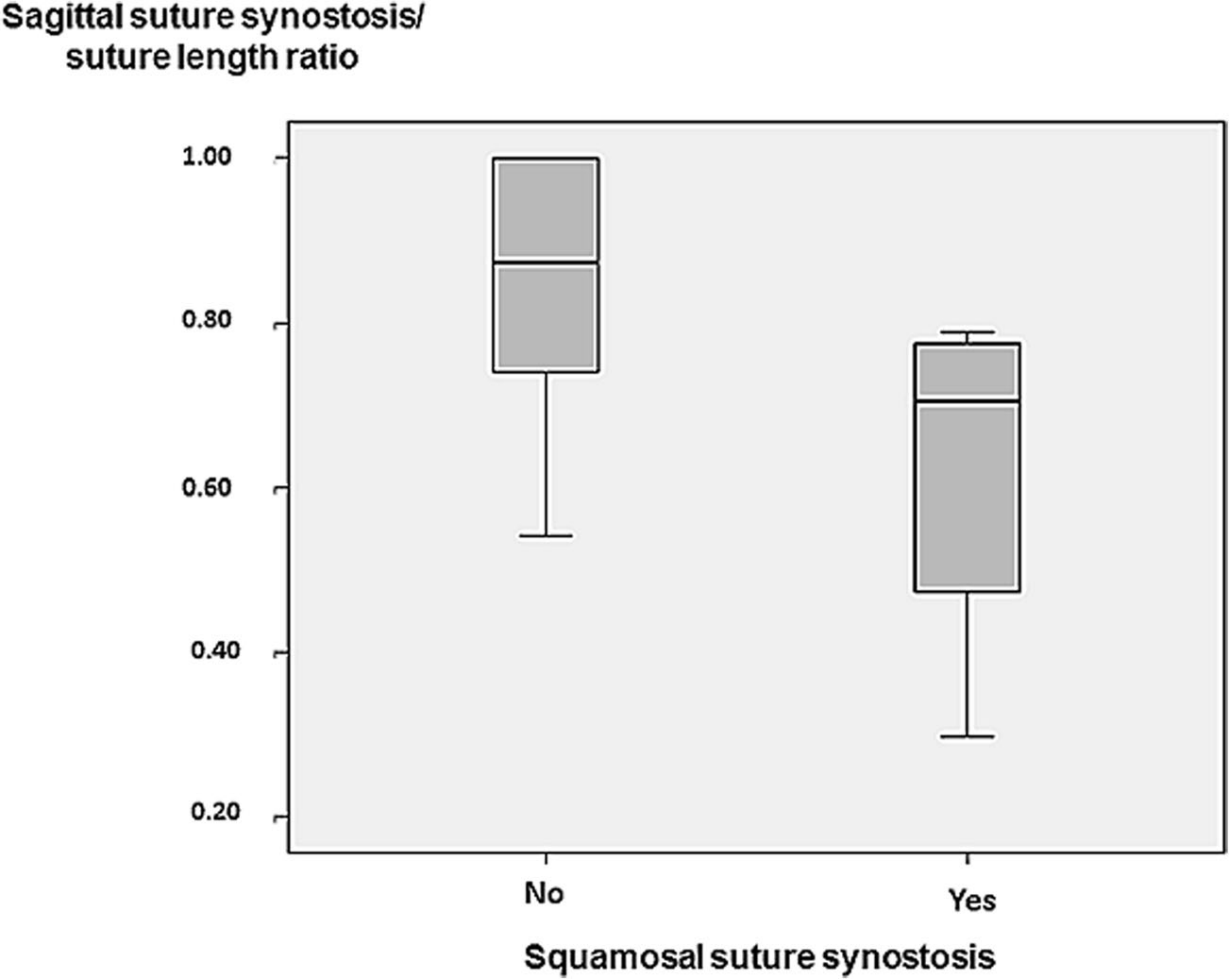
Box-plot displaying the sagittal suture synostosis length/suture length ratio (length of synostosis / total sagittal suture length × 100) in children with and without squamosal suture synostosis

All synostoses were unilateral, two were located on the right and two on the left side. Lengths of the synostoses varied from 4 to 27 mm. Children with SQS had shorter sagittal suture synostosis length ratio (length of synostosis / total sagittal suture length × 100) than those without SQS, *p* = 0.031 (Fig. 2).

In general, the patients with SQS were slightly younger (0.34 years, SD 0.11) than those without SQS (0.5 years, SD 0.05), but the difference was not significant (*p* = 0.285).

## Discussion

In our cohort of 34 non-syndromic sagittal synostosis patients, four children (11.7%) had an additional unilateral SQS. Eley et al. [2] have reported a lower 5% (7/144), and Murphy et al. [4] a higher 30% (3/30) incidence of SQS in non-syndromic sagittal synostosis. The patients of these previous studies with SQS have been older and their age range has been wider. The children with SQS in our study were slightly younger than those without SQS although the difference was not significant.

The additional SQS did not affect ICV. However, the children with additional SQS had shorter sagittal synostosis length ratio. It has been found that the length of the sagittal synostosis and SIV increase with age [3]. The ICV was larger also in the older children with SQS although length of sagittal synostosis increased.

Limitations of the present preliminary study include a small number of patients without a control group. A larger material would be needed for the evaluation of the possible growth restriction and asymmetry. Early infancy is essential to the surgeon who evaluates the child with craniosynostosis because this is the period of most rapid brain growth.

## Conclusion

The incidence of SQS in non-syndromic sagittal synostosis was 11.7% but SQS did not affect the ICV.
